# Accelerated Feature Selection via Discernibility Hashing: A Rough Set Approach

**DOI:** 10.3390/e27121222

**Published:** 2025-12-01

**Authors:** Sheng Luo, Linxiang Shi, Lin Chen, Xiaolin Cao

**Affiliations:** 1 School of Computer and Information, Shanghai Polytechnic University, Shanghai 201209, China; tjluosheng@gmail.com (S.L.); chenl@sspu.edu.cn (L.C.); xlcaosspu@163.com (X.C.); 2 Artificial Intelligence Institute, Shanghai Polytechnic University, Shanghai 201209, China

**Keywords:** discernibility matrix, rough sets, feature selection, discernibility hashing, attribute reduction

## Abstract

As a foundational analytical tool, the discernibility matrix plays a pivotal role in the systematic reduction of knowledge in rough set-based systems. Recent advancements in rough set theory have witnessed the proliferation of discernibility matrix-based knowledge reduction algorithms, with notable applications in classical, neighborhood, covering, and fuzzy rough set models. However, the quadratic growth of the discernibility matrix’s complexity (relative to domain size) imposes fundamental scalability limits, rendering it inefficient for real-world applications with massive datasets. To address this issue, we introduced a discernibility hashing strategy to limit the growth scale of the discernibility attributes and proposed a feature selection algorithm via discernibility hash based on rough set theory. First, on the premise of keeping the information of the original discernibility matrix unchanged, the method maps the discernibility attribute set of all objects to the storage unit through a hash function and records the number of collisions to construct a discernibility hash. By using this mapping, the two-dimensional matrix space can be reduced to a one-dimensional hash space, which greatly removes invalid and redundant elements. Secondly, based on the discernibility hash, an efficient knowledge reduction algorithm is proposed. The algorithm avoids invalid and redundant element attribute sets to participate in the knowledge reduction process and improves the efficiency of the algorithm. Finally, the experimental results show that the method is superior to the discernibility matrix method in terms of storage space and running time.

## 1. Introduction

The exponential growth of data volume exacerbates challenges for machine learning algorithms when processing high-dimensional data, including computational inefficiency, elevated storage costs, and the curse of dimensionality [1]. Feature selection is an efficient preprocessing method for high-dimensional data, generating a compact low-dimensional version of the data while retaining information for downstream machine learning models [2,3,4,5]. The objective of feature selection is to identify and retain task-relevant features while discarding irrelevant ones. To address the critical issue of feature correlation assessment, rough set theory [6] offers a quantitative method that evaluates the consistency between indiscernibility relationships induced by the selected feature set and the decision set, using upper and lower approximation spaces.

As a significant research area in rough set theory, the concept of feature selection (also known as attribute reduction) has received considerable attention [7,8,9] and has been widely applied in various fields of artificial intelligence such as classification learning [10], multi-label learning [11], clustering analysis [12], text analysis [13], etc. Similar to Principal Component Analysis (PCA) and Independent Component Analysis (ICA), attribute reduction aims to identify an optimal feature subset from the entire feature space of an information decision system while preserving equivalent discernibility to the original set [5]. In the context of rough sets, a reduct is also defined as a subset of condition attributes, which keeps the positive regions unchanged [6]. Based on the definition of reducts and the optimization strategies employed, attribute reduction methods can be broadly categorized into two groups [5,14]: One type of attribute reduction methods focuses on the work to extend the definition of reducts to fit various application circumstances, such as the probabilistic rough set model [15,16], variable precision rough set model [17,18], covering rough set model [19,20], fuzzy rough set model [21,22,23], and algebra viewpoint attribute reduction [24], etc. The other one focuses on finding quick calculation methods for reducts to enhance efficiency. From an efficient computing perspective, existing methods for obtaining a reduct of condition attributes can be broadly classified into two principal categories: information theoretic approaches that leverage heuristic measures [2,7,25,26,27] and discernibility matrix-based methods [3,6,8,9,28,29,30]. The first category employs entropy-based metrics and mutual information to guide feature selection, while the second utilizes discernibility matrices and equivalence class analysis to identify redundant features. Existing research has shown that the most significant factor influencing reducts is the definition and the corresponding calculation method [14]; thus, efficient computation of attribute reduction poses a pressing challenge that demands rigorous exploration. In this paper, we focus on the efficiency of the discernibility matrix-based feature selection method.

The discernibility matrix-based feature selection method operates by storing the discernibility attributes between domain objects in a discernibility matrix from which it subsequently derives the core and reduct with respect to the conditional attributes [31]. By employing matrix-based representation, this approach offers intuitive simplicity and has been widely adopted in attribute reduction computations. Unfortunately, the discernibility matrix also has some drawbacks, such as the space complexity and time complexity of the algorithm for constructing the discernibility matrix being $O(|U{|}^{2}\cdot |C|)$ as well as a large number of repetitive elements and invalid elements [8,32]. In response to the shortcomings of the discernibility matrix, some improvements have been made in the existing research. For instance, methods such as using the simplest discernibility matrix [8], binary tree structure [33], and tree structure [32,34] are employed to implement the calculation of the core and reduction. The storage structures adopted in these methods have improved the algorithm efficiency compared to the discernibility matrix. However, there are still some aspects that need to be enhanced for the subsequent feature selection algorithms, such as the mutual information calculation between candidate attributes and decision attributes, the complexity brought by the new structure in selecting candidate attributes, the difficulty in calculating the priority among attributes, etc. To simultaneously preserve the information entropy of the discernibility matrix while avoiding computational complexity in candidate attribute priority ranking, we propose a hash-based discernibility attribute set representation termed discernibility hash, enabling the construction of an accelerated feature selection algorithm. Through a combined hash mapping and conflict resolution mechanism, the discernibility hash intelligently filters repeated elements in the discernibility matrix, achieving both storage compression and computational efficiency. Due to the smaller size of stored elements brought by the transformation from 2D matrix representation to 1D hash representation of the discernibility attribute set, the theoretical time complexity and space complexity of the subsequent feature selection algorithm are improved. Therefore, according to the structural characteristics of discernibility hash representation, we propose an efficient feature selection algorithm based on discernibility hash representation, denoted FSDH. The experimental results show that our method not only reduces the time complexity and space complexity but also maintains the information entropy of the decision information system unchanged and achieves a selected feature subset that is consistent with the result of the methods based on discernibility matrix.

The main contributions of this paper are summarized as follows:

- We proposed a method of discernibility hash mapping for the discernibility attribute set of a decision information system, which solves the problem of excessive storage space complexity of the discernibility matrix.
- A fast feature selection algorithm based on discernibility hashing was proposed, which avoids the participation of duplicate elements and invalid elements in the set of discernibility attributes, thereby reducing time complexity and improving the performance of the algorithm.
- We also conducted experiments on the proposed algorithm to verify its effectiveness and efficiency. The experimental results show that our algorithm can achieve the same feature selection results as the discernibility matrix-based algorithm while significantly reducing the time and space complexity.

The rest of this paper is organized as follows. In Section 2, we briefly introduced some essential basic concepts and notations of the rough set theory. Section 3 mainly describes our proposed algorithms. The main contents include some basic concepts and definitions, the construction of discernibility hash, the design and analysis of the feature selection algorithm based on discernibility hash, etc. In Section 4, we introduce an example to demonstrate the results of the discernibility matrix and discernibility hash, analyze their differences, and discuss their structural connections. In Section 5, we designed an algorithm comparison experiment based on the UCI dataset and analyzed the experimental results in detail. Section 6 concludes this work with a comprehensive summary.

## 2. Preliminary

In this section, we will describe related preliminary notions for the classic Pawlak rough set theory. Furthermore, the research problem will be formulated with these concepts.

**Definition** **1** (Decision Information System)**.** *A decision information system [5,6] is formally defined as a four-tuple structure, S=(U,A,V,f), where*

*(1).*  *$U=\{x_{1},x_{2},\ldots ,x_{n}\}$ represents the object domain (universal set of all possible objects);*
*(2).*  *A is the attribute set composed of the conditional attribute C and the decision attribute D (i.e., A=C∪D);*
*(3).*  *V represents the value domain, expressed as $V=\cup_{a\in A}V_{a}$, where $V_{a}$ is the value set of attribute a;*
*(4).*  *f:U×A→V is a function which maps U×A to the value domain V.*

The following Table 1 shows an example of the decision information system. In the system, there are some calculate routines as follows, for example, $U=\{x_{1},x_{2},\ldots ,x_{7}\}$, C={a,b,c,d,e,f},D={g}, A=C∪D, $V_{a}=\left\{1,2\right\},V_{b}=\left\{0,1,2\right\}$, $V_{c}=\left\{0,1,2\right\}$, $V_{d}=\left\{0,1\right\}$, and $V_{e}=\left\{0,1,2\right\}$. For the function *f*, the following are still some computational examples: $f(x_{1},a)=1$, $f(x_{2},c)=0$, and $f(x_{3},e)=0$.

**Definition** **2** (Indiscernibility Relation)**.** *Given a subset R of attributes A(R⊆A), the indiscernibility relation [5] IND(R)⊆U×U is defined as follows:*

(1) IND(R)={(x,y)|∀x,y∈U,∀a∈R,f(x,a)=f(y,a)}

Let R={a,b}, the indiscernibility relation IND(R) partitions the objects into equivalence classes $\mathrm{IND}\left(R\right)=\{\left\{x_{1},x_{2}\right\},\left\{x_{3},x_{4}\right\},\left\{x_{6},x_{6},x_{7}\right\}\}$.

**Definition** **3** (Reduct)**.** *Given a decision information system S, the attribute set R⊆A is defined as a reduct [6], if R satisfies the following conditions:*

*(1).*  IND(R)=IND(A);
*(2).*  IND(R−{a})≠IND(A), ∀a∈R.

**Definition** **4** (Core)**.** *The core of a decision information system S with respect to attribute set A is defined as the intersection of all its reducts, i.e.,*

(2) $$Core\left(A\right)=\underset{R\in Reduct(A)}{⋂}R$$

*where Reduct(A) stands for the set of all possible reducts of attribute set A.*

**Definition** **5** (Discernibility Matrices)**.** *Let S be a decision information system, then the discernibility matrix [31] $M=(m_{ij})$ of S is defined as a set, i.e.,*

(3) $$m_{ij}=\left\{\begin{array}{cc} \{a\}, & \forall a\in C,f\left(x_{i},a\right)\neq f\left(x_{j},a\right)\wedge f\left(x_{i},D\right)\neq f\left(x_{j},D\right);\\ \emptyset , & \mathrm{otherwise}. \end{array}\right.$$

*where $m_{ij}$ denotes an element of the matrix M with the condition 1≤i,j≤n.*

The meaning of the entry $M_{ij}$ is that the object pair $\left(x_{i},x_{j}\right)$ can be distinguished by any element in the attribute set $M_{ij}$. In other words, the objects $x_{i}$ and $x_{j}$ are discerned precisely when the condition $m_{ij}\neq \emptyset$ is satisfied. It is obvious that discernibility matrix *M* is symmetric, i.e., $m_{ij}=m_{ji}$ and $m_{ii}=\emptyset ,\forall i,j\in \left[1,n\right]$. Therefore, to minimize storage redundancy, we can retain only one triangular portion (either lower or upper) of the symmetric matrix, discarding the redundant counterpart. This approach preserves all essential information while reducing storage requirements by half.

**Theorem** **1.** *Given a decision information system S and its discernibility matrix M, an attribute set R is a reduct if and only if [8]*

*(1).*  *$\forall (x_{i},x_{j})\in U\times U$ and $m_{ij}\neq \emptyset ,\left[R\cap m_{ij}\neq \emptyset \right]$;*
*(2).*  *$\forall a\in R,\exists \left(x_{i},x_{j}\right)\in U\times U,\left[m_{ij}\neq \emptyset \right]$ and $\left[\left(R-\left\{a\right\}\right)\cap m_{ij}=\emptyset \right]$.*

The proposition above enables the validation of attribute subsets as potential reducts by leveraging the discernibility matrix of a decision information system. However, the problem of how to construct a reduct based on the discernibility matrix still remains unsolved.

## 3. Proposed Method

As defined in the previous section’s discernibility matrix formulation, the time complexity and the space complexity of the algorithm for constructing the discernibility matrix are $O(|U{|}^{2}\times |C|)$. It is not difficult to find that there are redundant calculation elements [8,9] in the discernibility matrix. By eliminating these redundant elements, storage space can be significantly compressed, which in turn reduces the computational frequency of the attribute reduction algorithm and enhances overall efficiency. Moreover, as the object cardinality of the decision information system grows, the probability of repeated occurrences of the discernibility attribute set within the matrix proportionally increases. To mitigate redundant computations and storage overhead, we introduce a novel hash-based storage structure and a corresponding accelerated attribute reduction algorithm.

**Definition** **6** (Ordered Discernibility Attributes)**.** *Given a decision information system S, the discernibility attribute of an object pair $(x_{i},x_{j}),1\leq i,j\leq n$, is formally defined as follows:*

(4) $$h_{k}=\left\{\begin{array}{cc} \varphi (\{a\}), & \forall a\in C,f\left(x_{i},a\right)\neq f\left(x_{j},a\right)\wedge f\left(x_{i},D\right)\neq f\left(x_{j},D\right);\\ \emptyset , & otherwise. \end{array}\right.$$

*where φ(·) is a sorting strategy that arranges the elements of the set {a} in ascending (or descending) order, and k is a number that is much smaller than $n^{2}$ and it depends on the repetition rate of the discernibility attribute set.*

For instance, suppose there are the following set of discernibility attributes,

{a,b,d},{c,a},{b,d,a},{a,b,c}

then, we can obtain the ordered discernibility attributes

$$\begin{array}{cc} \varphi (\{a,b,d\}) & =\;\{a,b,d\}\\ \varphi (\{c,a\}) & =\;\{a,c\}\\ \varphi (\{b,d,a\}) & =\;\{a,b,d\}\\ \varphi (\{a,b,c\}) & =\;\{a,b,c\} \end{array}$$

It can be seen that the first line of the above equation is equivalent to the third line. This also reveals the implicit relationship between *k* and i,j, that is, $h_{k}$ is equal to all the $m_{ij}$ elements that have the same ordered discernibility attributes.

**Definition** **7** (Discernibility Hash)**.** *Given a decision information system S and its ordered discernibility attributes $h_{k}\left[h_{k}=\varphi \left(m_{ij}\right),\forall x_{i},x_{j}\in U\right]$, the discernibility hash function is defined as follows:*

(5) $$\delta \left(h_{k}\right)={\left(h_{k}\right)}_{r}$$

*where r represents the encoding cardinality of the key. All the discernibility attributes of the decision information system S are calculated by the discernibility hash function to form a hash table called the discernibility hash.*

Consider a decision information system *S* containing discernibility attributes, for example,   

$$h_{1}=\left\{a,c\right\},h_{3}=\left\{a,b,c\right\}\mathrm{and}h_{4}=\left\{b,c\right\}.$$

The corresponding hash values are computed, respectively, as

$$\delta \left(h_{1}\right)=101,\delta \left(h_{3}\right)=111,\mathrm{and}\delta \left(h_{4}\right)=011$$

by setting the parameter r=2. To simplify the problem, it is also possible to simply adopt the identity function as the hash function, i.e., remove the digit ’0’ from the binary encoding, and then replace the digit ’1’ directly with the corresponding attribute name. Therefore, we can obtain the keys of the hash table as follows:

$$\delta \left(h_{1}\right)=ac,\delta \left(h_{3}\right)=abc,\mathrm{and}\delta \left(h_{4}\right)=bc$$

Unlike the discernibility matrix that stores all discernibility attributes explicitly, the hash-based approach maps key values directly to storage locations through deterministic hash functions; while both storage mechanisms exhibit comparable access time performance, the hash-based approach demonstrates superior space efficiency by effectively compressing redundant elements and empty slots inherent in the discernibility matrix. In hash-based storage systems, collisions inevitably occur when distinct keys share identical hash values. These collisions directly correspond to redundant elements in the discernibility matrix, where multiple object pairs may yield identical discernibility attributes. Therefore, the number of hash value collisions directly quantifies the redundancy in stored discernibility attributes, where each collision indicates a repeated entry in the discernibility matrix. For large-scale datasets, a limited set of attributes will inevitably produce a large number of repeated discernibility attribute subsets. Removing redundancy will optimize the calculation, which can greatly accelerate the calculation of attribute reduction.

**Theorem** **2.** *Given a decision information system S and its discernibility hash H, an attribute set R is a reduct if and only if*

*(1).*  *∀h∈H,R∩h≠∅;*
*(2).*  *∀a∈R,∃h∈H,(R−{a})∩h=∅.*

**Proof.**  Let $\mathrm{IND}\left(C\right)=\left\{C_{1},C_{2},\ldots ,C_{l}\right\}$ and $\mathrm{IND}\left(R\right)=\left\{R_{1},R_{2},\ldots ,R_{l}\right\}$, where *l* denotes the cardinality of the equivalence classes induced by the conditional attribute set *C* and the decision attribute *D*. For any non-empty set h∈H, it indicates that

(6) $$\left\{a\right\}=\delta^{-1}\left(\varphi^{-1}\left(h\right)\right)$$

Then, there exists $x_{i},x_{j}$ such that $f\left(x_{i},a\right)\neq f\left(x_{j},a\right)\wedge f\left(x_{i},D\right)\neq f\left(x_{j},D\right)$. That is, the object pair $x_{i},x_{j}$ belongs to different partitions, i.e.,

(7) $$\mathrm{if}\;x_{i}\in C_{i}\wedge x_{j}\in C_{j}.\;\mathrm{then}\;C_{i}\neq C_{j}.$$

If R∩h=∅, then we can claim that the object pair $x_{i},x_{j}$ belongs to the identical partition induced by the attribute set *R*, which is contradictory to Equation (7)., i.e.,

IND(C)≠IND(R)

As a result, Condition 1 is satisfied. Suppose that after removing element *a* from *R*, there exists no h∈H such that (R∖{a})∩h=∅). This implies (R∖{a}) remains a reduct, which contradicts the definition of reducts. Therefore, Condition 2 must hold.    □

Condition 1 establishes that *R* is sufficient for distinguishing all discernible object pairs. It is easy to see that the union of all the elements of discernibility hash *H* must satisfy Condition 1. Condition 2 shows whether each element in *R* can be removed.

**Theorem** **3.** *The discernibility hash and the discernibility matrix derived from the same information system are informationally equivalent, preserving identical sets of discernibility attributes despite their distinct storage representations.*

**Proof.**  The probability that the element $m_{ij}\left(1\leq i,j\leq n\right)$ of the arbitrary discernibility matrix *M* is as follows:

(8) $$p_{\left\{attrs\right\}}=\frac{2\times \#(\{attrs\})}{n(n-1)}$$

where #(·) is a function to calculate the occurrence frequency of attribute set $\left\{attrs\right\}≜m_{ij}$ within the discernibility matrix *M*, and $n,\frac{n(n-1)}{2}$ are the total number of objects and entries in the decision information system *S*, respectively. Then, the information entropy of the matrix *M* is as follows:

(9) $$\mathrm{entropy}\left(M\right)=\sum_{\left\{attrs\right\}}-p_{\left\{attrs\right\}}\cdot \log\left(p_{\left\{attrs\right\}}\right)$$

For any ordered discernibility attribute $h_{k}\in H$, the probability of it appearing in the discernibility hash is

(10) $$p_{\mathrm{key}}=\frac{\mathrm{freq}(\delta (\mathrm{key}))\times 2}{n(n-1)}$$

where $\mathrm{key}≜h_{k}\left(1\leq k\leq n\right)$, freq(·) computes the frequency of the key appearing in discernibility hash *H*. For each discernibility attribute pair $(m_{ij},h_{k})\in M\times H$, the following equation necessarily holds:

(11) $$\#\left(m_{ij}\right)=\mathrm{freq}\left(\delta \left(h_{k}\right)\right),\;\mathrm{if}\forall 1\leq i,j,k\leq n,\mathrm{sorted}\left(m_{ij}\right)=h_{k}.$$

In other words, any discernibility attribute set appears the same number of times in the discernibility matrix and the discernibility hash, regardless of the type of storage structure. Therefore, the following equation holds true:

(12) entropy(M)=entropy(H).

   □

As a consequence, after using discernibility hash representation, the amount of information entropy in the system is equal to that of the discernibility matrix. The two approaches are logically equivalent, differing solely in their storage architecture (e.g., adjacency list vs. hash table), with no information loss during representation. Obviously, by taking advantage of hash collisions, we can significantly reduce the size of the discernibility attributes set, thereby lowering the complexity of the subsequent processing algorithm.

### Efficient Knowledge Reduction Algorithm

The knowledge reduction algorithm is able to divided into two stages. The initial stage systematically computes all discernibility attributes for object pairs in the decision information system, subsequently storing them in a target hash table. The final stage employs the discernibility hash strategy to construct the reduct of the decision information system, thereby optimizing both computational efficiency and storage utilization. The construction algorithm of the discernibility hash table and the corresponding fast knowledge reduction algorithm are presented below.

An important criterion for a hash function, Equation (5), is that it must ensure that different keys are mapped to different addresses, while the same key must be mapped to the same address. The purpose of the discernibility hash is to utilize the hash conflicts to compress the repeated occurrence of the same key in the discernibility. If different sets of discernibility attributes are mapped to the same storage location, it will cause calculation difficulties for subsequent knowledge reduction. This is the most distinctive feature that sets Equation (5) apart from others. Obviously, the time complexity of Algorithm 1 is the same as that of the discernibility matrix-based algorithm, both being $O(n^{2})$.
**Algorithm 1** Discernibility hash construction algorithm**Input:** 
*S*: the decision information system; δ(·): the discernibility hash function.**Output:** 
*H*: the discernibility hash table of *S*.1:**for** i=1,2,…,N **do**2:    **for** j=1,2,…,i **do**3:        $m_{ij}\leftarrow$ Equation (3);4:        key← Equation (4);5:        $loc_{key}\leftarrow$ Equation (5): calculate the mapping addresses of the discernibility attributes;6:        insert $loc_{hk}$ into the target hash table *H* and track the number of collision;7:    **end for**8:**end for**9:return the discernibility hash *H*.

Based on the output of Algorithm 1, we propose an efficient feature selection algorithm based on discernibility hash strategy, termed FSDH, in Algorithm 2.
**Algorithm 2** Efficient feature selection algorithm based on discernibility hash, FSDH**Input:** 
*H*: the discernibility hash *H*.**Output:** 
CORE(A): the core of the attribute set *A*; RED(A): a reduct of the attribute set *A*.1:attr_freq← the frequency of all attributes of the difference hash;2:CORE(A)←∅;3:**for** key in *H* **do**4:    **if** len(key)==1 and key not in CORE(A) **then**5:        add key to the set CORE(A);6:    **end if**7:**end for**8:RED(A)←CORE(A);9:**repeat**10:    a←max_item(attr_freq); //select the highest frequency item from attr_freq11:    add *a* to the set RED(A);12:    remove all keys containing *a* from the discernibility hash *H*;13:**until** {RED(A) meets the reduction criteria (Definition 3)}14:return CORE(A),RED(A);

The algorithm JohnsonReduct [32] is an attribute reduction algorithm that utilizes the greedy strategy based on the discernibility matrix. The difference between JohnsonReduct and FSDH lies solely in their storage representation of the identical discernibility attribute set: that is, JohnsonReduct employs a discernibility matrix, whereas FSDH utilizes a hash table.

**Theorem** **4.** *FSDH and JohnsonReduct have the same output result, and the time upper bound of FSDH is that of JohnsonReduct, while the lower bound is determined by the storage compression rate ρ (Equation (17)).*

**Proof.** 
Suppose the set of the discernibility attribute set is As, the discernibility matrix is $M=(m_{ij})$, and the discernibility hash is $H=[h:v_{h}]$. According to Theorem 3, for any discernibility attribute set s∈As, it can be concluded that

(13) $$\begin{array}{cc} \forall i,j\in [1,n],\; & \exists m_{ij}\wedge \mathrm{freq}\left(m_{ij}\right)=\mathrm{freq}\left(s\right)\\ \forall k\in [1,|H|],\; & \exists h_{k}=\delta \left(\varphi \left(m_{ij}\right)\right)\wedge v_{h_{k}}=\mathrm{freq}\left(s\right) \end{array}$$

where freq(s) is a counting function, and $v_{h_{k}}$ stands for the value of the key $h_{k}$. Consequently, since the set of discernibility attributes stored in matrix and hash formats are statistically equivalent, the outputs of the two algorithms are consistent.

Algorithm 2 demonstrates that the discernibility matrix and hash differ exclusively in their first and 12th rows. Given identical storage structures, these rows exhibit equivalent time and space complexities. If the discernibility matrix M is used, the time complexity of the first and 12th rows is as follows:

(14) $$\begin{array}{cc} T(n) & =\sum_{i=1}^{n}\sum_{j=1}^{i}\sum_{k=1}^{\left|C\right|}1\\ \; & =\left|C\right|\sum_{i=1}^{n}i=\left|C\right|\frac{n(n+1)}{2} \end{array}$$

Therefore, the time complexity is $O(n^{2})$ when |C|<n, otherwise it is $O(n^{3})$. For the discernibility hash H, since the discernibility attribute set has been encoded, it can be directly mapped to the physical address. Hence, the operation of determining whether a candidate attribute belongs to a discernibility attribute set can be directly performed using the encoding value through subtraction, with a time complexity of O(1). Then, the time complexity of the discernibility hash H is

(15) $$F\left(n\right)=\sum_{i=1}^{\left|H\right|}1=O\left(|H|\right)$$

where |H| is the length of hash table H, and $\left|H\right|=\frac{n^{2}}{2}\rho$ (ρ represents the compression rate, with a value range of [0, 1]). In conclusion, the following inequalities hold:

(16) $$O\left(n^{2}\cdot \frac{\rho }{2}\right)\leq F\left(n\right)\leq T\left(n\right)$$

That is, the upper bound of the time complexity of FSDH is JohnsonReduct, while the lower bound is determined by the compression rate ρ. □

The computational bottleneck of the feature selection algorithm primarily arises from traversing the discernibility matrix, where repeated attribute comparisons dominate the runtime complexity. The proposed algorithm achieves significant space complexity reduction, transitioning from $O(n^{2}\times |C|)$ in the traditional knowledge reduction algorithm to O(|H|) in our hash-based approach, where $\left|H\right|\approx \frac{n^{2}}{2}\cdot \rho$. This improvement is particularly notable as $\frac{n^{2}}{2}\cdot \rho \ll n^{2}\times \left|C\right|$ for large-scale datasets. In the subsequent experimental section, the algorithm’s results on the UCI dataset confirmed the correctness of this statement.

## 4. Case Study

This section uses an example to analyze the differences between the discernibility hash and the discernibility matrix. We adopt the benchmark dataset [32], referred to as Toy1, as our target decision information system for evaluation. The dataset Toy1, as presented in Table 2, comprises four conditional attributes (columns a−d), one decision attribute (column *e*), and seven objects.

Applying Equation (3) to the Toy1 dataset yields the discernibility matrix presented in Table 3, where each entry $m_{ij}$ quantifies the attribute-level dissimilarity between objects $x_{i}$ and $x_{j}$. As demonstrated in Table 3, the discernibility matrix consists of 21 elements, including 6 empty sets and repeated sets of discernibility attributes. Among these, the empty sets can be completely ignored, while the repeated attribute sets can be compressed. Obviously, the discernibility matrix exhibits significant redundancy in stored attribute sets. By employing hash-based compression techniques, these repetitive elements can be systematically eliminated, achieving dual objectives: (1) reducing space complexity from $O(n^{2})$ to O(|H|) and (2) accelerating subsequent knowledge reduction operations through streamlined data access.

The discernibility hash table for the dataset Toy1, constructed using Algorithm 1, is presented in Table 4. This table systematically maps discernibility attributes to their corresponding hash values, demonstrating the algorithm’s efficiency in reducing storage redundancy. The symbol ρ in the table stands for the storage compression rate which is calculated by the following equation:

(17) $$\rho =\frac{\mathrm{the}\mathrm{total}\mathrm{number}\mathrm{of}\mathrm{unique}\mathrm{set}\mathrm{of}\mathrm{discernibility}\mathrm{attributes}}{\mathrm{the}\mathrm{total}\mathrm{number}\mathrm{of}\mathrm{the}\mathrm{set}\mathrm{of}\mathrm{discernibility}\mathrm{attributes}}$$

The compression rate ρ reflects the degree of conflict in the discernibility hash table. The higher the degree of conflict, the more sets of discernibility attributes are repeated, and thus the smaller the compressible space will be.

**Table 4:** The discernibility hash table of the dataset Toy1.

Key	Item Frequency
{ab}	2
{abc}	1
{abcd}	4
{abd}	1
{b}	1
{bc}	1
{bcd}	2
{cd}	3
compression rate ρ:	38.1%

By comparing Table 3 and Table 4, it can be seen that, compared with the discernibility matrix, the size of the discernibility hash has decreased from 21 to 8, and the compression storage rate is 8/21≈38.1%. In other words, the discernibility hash method achieves a 38.1% reduction in storage space compared to the discernibility matrix, as it eliminates the need to store and compute 61.8% of duplicate entries in subsequent steps. Therefore, the advantage of discernibility hash *H* becomes evident; that is, while keeping the system’s information entropy unchanged, the storage space has been effectively compressed.

## 5. Experiments

The empirical study of the FSDH is given in this section. We first setup the experiments by introducing the datasets. Then we conduct a comprehensive performance evaluation by analyzing both time complexity and space usages through rigorous empirical testing.

### 5.1. Experimental Setup

The experimental platform utilized in this study comprised a Core i7-8550U CPU (1.80 GHz base clock) with 16 GB DDR4 RAM, ensuring sufficient computational capacity for all tests. To test the performance of our method, we employed seven benchmark datasets for evaluation: six from the UCI Machine Learning Repository (Weather, Contact-lenses, Zoo, Breast-cancer, Vote, and Tictactoe) and one synthetic dataset (Toy1). Table 5 presents comprehensive descriptive statistics for the seven benchmark datasets, including sample sizes, attribute sizes, and class sizes.

In order to verify the performance differences between discernibility matrices and discernibility hash tables, we chose the algorithm JohnsonReduct [32], which uses the same heuristic strategy as FSDH, for comparison.

### 5.2. Performance Analysis of FSDH

Table 6 presents a comparative analysis of temporal and spatial complexity across seven benchmark datasets, with execution time (in seconds) and storage consumption (in unit amounts) quantitatively evaluated under uniform experimental conditions. In the table, the column “Count of units” represents the number of the set of discernibility attributes generated and stored by the algorithm JohnsonReduct and FSDH, respectively, and the column “Time (s) ” stands for the time consumed by the algorithm, measured in seconds. Furthermore, the meaning of “Time rate”, denoted as τ in the table, is as shown in the following equation:

(18) $$\tau =\frac{\mathrm{Executation}\mathrm{time}\mathrm{of}\mathrm{FSDH}}{\mathrm{Executation}\mathrm{time}\mathrm{of}\mathrm{JohnsonReduct}}$$

The ρ in the table is consistent with Equation (17), representing the compression degree of all storage units.

**Table 6:** Experimental results of the UCI datasets.

	JohnsonReduct		FSDH		Time Rate (τ)	Compression Rate (ρ)
	Count of Units	Time (s)	Count of Units	Time (s)		
Toy1	21	4.96 × 10^−4^	8	7.73 × 10^−5^	15.59%	38.10%
Weather	91	8.38 × 10^−4^	13	9.04 × 10^−5^	10.79%	14.29%
Contact-lenses	276	3.53 × 10^−4^	15	1.05 × 10^−4^	29.76%	5.43%
Zoo	5050	4.91 × 10^−2^	956	2.95 × 10^−3^	6.00%	18.93%
Breast	40,755	2.93 × 10^−1^	488	7.30 × 10^−3^	2.50%	1.20%
Vote	94,395	7.85 × 10^0^	10,548	1.06 × 10^0^	13.53%	11.17%
Tictactoe	458,403	2.27 × 10^1^	501	7.15 × 10^−3^	0.03%	0.11%

As can be seen from Table 6, the algorithm FSDH that based on discernibility hash shows significant improvement in efficiency compared to the algorithm JohnsonReduct, which based on discernibility matrix, both in terms of time and space cost. In these datasets, the algorithm demonstrates its most significant improvement in dataset Tictactoe, where the entry size of the discernibility matrix is reduced from 458,403 to 501 (achieving a compression rate of 0.11%). As a result, the execution time of the subsequent feature selection algorithm decreases to merely 0.03% of the original algorithm’s time. For dataset Contact-lenses, despite having the lowest compression rate among the datasets, the storage units still achieve a compression rate of 29.76%, which constitutes a substantial improvement for the discernibility matrix.

**Figure 1:**
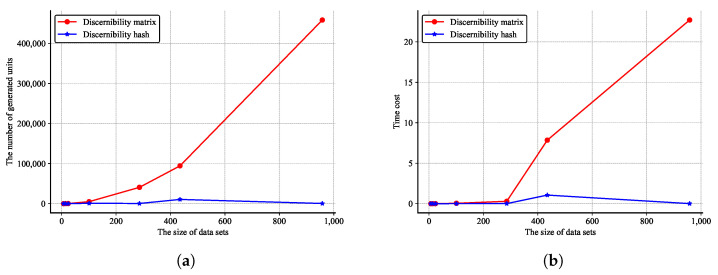
Comparison of algorithm complexity. (**a**) The comparison of storage unit amount. (**b**) Execution time comparison.

Figure 1a shows the comparison of storage units produced by the two structures on datasets of different sizes. As the size of the dataset increases from 7 to 958, the number of discernibility matrix cells also grows accordingly at a rate of $O(n^{2})$, while the growth rate of hash cells is approximately linear, at O(n). It indicates that as the sample size increases, the repetition rate of the discernibility attributes also shows an increasing trend. Therefore, a large number of repetitive units in the discernibility matrices are completely unnecessary to retain. After deletion, it can save computing resources and avoid redundant calculations. Avoiding repetition is precisely what discernibility hashing is good at. By using the hashing mechanism, conflicts can be utilized to filter out duplicate elements, without any loss of the system’s information entropy, achieving the goal of simultaneously saving computing time and storage space.

The removal of repetitive elements via filtering directly contributes to a marked increase in the computational efficiency of feature selection algorithms. Figure 1b demonstrates the performance of discernibility matrix- and discernibility hash-based approaches across datasets of varying scales, revealing their distinct impacts on feature selection efficiency. From lines 1 and 12 of Algorithm 2, it can be seen that the time complexity of this algorithm is closely related to the size of all unique discernibility attributes. Therefore, the size of the unique discernibility attributes is the key factor determining the efficiency of the feature selection algorithms. The redundant storage can prove to be completely unnecessary and increase the complexity of the algorithm. This can be seen form Figure 1b, especially when the sample data reaches a certain level, for example, 400 in the figure, this characteristic becomes very significant.

In summary, the feature selection algorithm based on discernibility hashing not only reduces redundant storage units without losing the system information entropy but also significantly improves the execution time compared to the discernibility matrix based feature selection algorithms.

### 5.3. Comparison with Other Rough Set Feature Selection Methods

To evaluate our algorithm’s performance, we compared it against three widely adopted and representative methods, i.e., entropy-based attribute reduction (EBAR) [35], quick attribute reduction algorithm (QAR) [36], and Pawlak’s attribute reduction algorithm (Pawlak) [37]. Performance evaluation still employs the seven UCI datasets from the previous section, with execution speed (total runtime per dataset) as the primary metric. Table 7 presents the runtime results (in seconds) of all algorithms, with the optimal result highlighted in bold.

As can be seen from the table above, among all the datasets, our FSDH method has the highest execution efficiency, except for the dataset Vote. While FSDH showed lower efficiency than Pawlak on the Vote dataset, it still outperformed the other two methods. The time spent by FSDH was only 4.04% of the method QAR and 61.61% of that of the method EBAR. These results indicate that the feature selection method based on differential hashing demonstrates a significantly greater improvement compared to other feature selection algorithms. The main reason for the effective improvement lies in the fact that our method, by filtering out unnecessary empty elements and compressing duplicate elements, has saved a significant amount of storage space. Therefore, when conducting the traversal and comparison of all the difference attribute sets, the scope is significantly reduced, thereby minimizing the redundant comparison time.

In summary, the FSDH method which has the capability to remove empty elements and compress duplicate elements in a discernibility attribute set, could save a significant amount of time in conducting searches for discernibility attributes, so that it is more conducive to achieving a better performance.

## 6. Conclusions

Due to the redundant storage units, it is a challenging task to effectively and robustly discover principal components of a decision information system, especially when the size of the training set reaches a certain level. In this work, we proposed a novel representation for discernibility attributes, i.e., discernibility hash, and developed an efficient feature selection algorithm to choose the most important feature of the decision information system for the subsequent tasks. Experiments show that our method is superior to the discernibility matrix-based methods.

Several aspects of the new method are worth investigating in further depth, including how to compress the discernibility hash, feature selection strategies and how to handle the numerical feature, etc. In the future, our work will be focused on the compression of discernibility hash, because a good data representation will be able to reduce algorithm complexity and also improve algorithm efficiency.

## Figures and Tables

**Table 1:** A decision information system.

Obj.	a	b	c	d	e	f	g
$x_{1}$	1	0	1	1	2	0	1
$x_{2}$	1	0	0	0	2	1	1
$x_{3}$	1	2	0	0	0	1	2
$x_{4}$	1	2	2	1	0	0	0
$x_{5}$	2	1	0	0	1	2	2
$x_{6}$	2	1	1	0	1	0	2
$x_{7}$	2	1	2	1	1	0	1

**Table 2:** Example dataset Toy1.

Obj.	a	b	c	d	e
$x_{1}$	1	0	1	1	1
$x_{2}$	1	0	0	0	1
$x_{3}$	1	2	0	0	2
$x_{4}$	1	2	2	1	0
$x_{5}$	2	1	0	0	2
$x_{6}$	2	1	1	0	2
$x_{7}$	2	1	2	1	1

**Table 3:** The discernibility matrix of the dataset Toy1.

	$x_{1}$	$x_{2}$	$x_{3}$	$x_{4}$	$x_{5}$	$x_{6}$
$x_{2}$	∅					
$x_{3}$	{bcd}	{b}				
$x_{4}$	{bc}	{bcd}	{cd}			
$x_{5}$	{abcd}	{ab}	∅	{abcd}		
$x_{6}$	{abd}	{abc}	∅	{abcd}	∅	
$x_{7}$	∅	∅	{abcd}	{ab}	{cd}	{cd}

**Table 5:** Selected datasets.

Name	Abbreviation	Instances	Attributes	Classes
toy1	D1	7	4	3
weather	D2	14	4	2
contact-lenses	D3	24	4	3
zoo	D4	101	17	7
breast-cancer	D5	286	9	2
vote	D6	435	16	2
tictactoe	D7	958	9	2

**Table 7:** Comparison of execution time with rough set-based feature selection methods.

	JohnsonReduct	FSDH	Pawlak	QAR	EBAR
toy1	4.96 × 10^−4^	**7.73 × 10^−5^**	2.03 × 10^−2^	6.01 × 10^−2^	2.36 × 10^−2^
weather	8.38 × 10^−4^	**9.04 × 10^−5^**	3.46 × 10^−2^	8.67 × 10^−2^	3.87 × 10^−2^
contact-lenses	3.53 × 10^−4^	**1.05 × 10^−4^**	2.89 × 10^−2^	1.22 × 10^−1^	5.89 × 10^−2^
zoo	4.91 × 10^−2^	**2.95 × 10^−3^**	1.05 × 10^−1^	1.50 × 10^0^	1.84 × 10^−1^
breast	2.93 × 10^−1^	**7.30 × 10^−3^**	2.45 × 10^0^	5.58 × 10^0^	9.20 × 10^−1^
vote	7.85 × 10^0^	1.06 × 10^0^	**1.77 × 10^−1^**	2.63 × 10^1^	1.72 × 10^0^
tictactoe	2.27 × 10^1^	**7.15 × 10^−3^**	4.30 × 10^−1^	7.55 × 10^0^	1.49 × 10^0^

## Data Availability

The original contributions presented in this study are included in the article. Further inquiries can be directed to the corresponding author.
